# Buprenorphine-naloxone in chronic pain: Overcoming stigma for safer opioid management

**DOI:** 10.1177/17151635231214508

**Published:** 2023-11-28

**Authors:** Naomi Steenhof, Karen Ng

**Affiliations:** Leslie Dan Faculty of Pharmacy (Steenhof), University of Toronto and Women’s College Hospital, Toronto Academic Pain Medicine Institute (Ng)

## Chronic pain in Canada

One in 5 Canadians lives with chronic pain, and as our population ages, the prevalence and impact of this disease are expected to grow.^1^ A multimodal combination of pharmacologic and nonpharmacologic strategies delivered through an interdisciplinary team is essential to chronic pain management.^2^ This level of evidence-based care, rooted in a biopsychosocial approach, requires substantial resources, and access remains a challenge. The availability of nonpharmacologic approaches such as physiotherapy and psychological counselling is impeded by coverage problems and scarcity of trained health care personnel in chronic pain, resulting in a heavy reliance on medications, particularly opioids. While opioids may be an appropriate option for some patients, many Canadians are being prescribed high doses over many years, which presents a considerable risk of harm for what might be only a short-term benefit.^3^

## The role of the pharmacist

Community pharmacists are accessible health care providers and well positioned to help patients optimize their opioid therapies in chronic pain. Pharmacists should be supported to develop skills to initiate the rotation, tapering and discontinuation of opioids for chronic pain to reduce the risk of opioid-related harms.^4^ However, in some situations, tapering and/or discontinuation of full-agonist opioids may be clinically challenging or not in the best interest of the patient. As a strategy to provide safer chronic pain management for patients who are dependent on opioids, buprenorphine-naloxone (bup-nal; Suboxone) is being prescribed off-label for the management of chronic pain.^5,6^

We (the authors) are pharmacists working in specialized tertiary academic chronic pain clinics that provide comprehensive medication management to patients who suffer from chronic pain. We wanted to share our clinical experiences, including some of the challenges that we have encountered in using bup-nal as a management option for our patients. We hope that this will encourage Canadian pharmacists to build the knowledge and skills to better support patients who choose bup-nal as an option for chronic pain management.

## The role of bup-nal in chronic pain management

Buprenorphine is a semisynthetic partial agonist opioid that was developed as an analgesic in the 1970s. While buprenorphine in the form of a patch (BuTrans) is indicated for the management of pain in adults, buprenorphine in combination with naloxone sublingually (Suboxone) is indicated only for “substitution treatment in adults with problematic opioid drug dependence.”^7^ Of note, the naloxone is added to the formulation to discourage intravenous use; however, when taken sublingually, it has no major clinical effect.

Buprenorphine is an ideal drug for pain management as it has a high-binding affinity and a slow rate of dissociation from the mu-opioid receptor, which produces a longer duration of action compared with other opioids.^8^ From an efficacy perspective, although buprenorphine is a partial mu-opioid agonist, human brain positron emission tomography scans have shown full analgesia can be achieved even when buprenorphine occupies less than 100% of the receptors.^9^ Buprenorphine’s clinical analgesic effect is likely due to its multimechanistic pharmacology.

In our experience, patients with chronic pain who are rotated to bup-nal see a decrease of 1 to 2 points on the verbal pain scale; however, the tangible benefit is seen in the improvement of function, mood, sleep, relationships with other people and ability to do day-to-day work.^9^ Achieving functional goals in chronic pain management is one of the reasons why many of our patients choose to stay on bup-nal for years.

From a safety perspective, buprenorphine has many advantages. Because it is a partial agonist, there is a low risk of opioid poisoning and a ceiling effect on respiratory depression.^5^ There is also a lower risk of sedation, and patients report less cognitive impairment.^8,10^ The risk of opioid-induced hyperalgesia and tolerance development is lowered as well.^11^ Once stabilized on bup-nal, patients also have little to no opioid withdrawal-mediated pain due to the long half-life of buprenorphine, which contributes to stability and improvement in function.

## Challenges due to stigma

Unfortunately, most of the challenges that we have seen with bup-nal have not occurred due to a lack of drug efficacy. They have, instead, occurred due to stigma surrounding bup-nal. This has been difficult for us as pharmacists, as our patients who are prescribed the BuTrans patch, which contains the same molecule (albeit at a lower dose) and has the same mechanism of action, do not report experiencing this stigma.

Our patients rotated to bup-nal have reported that their primary care providers are not comfortable or refuse to continue prescribing even after they have been stabilized at our pain clinics on low to moderate doses (2 mg/0.5 mg to 16 mg/4 mg per day). They also experience discrimination from pharmacists and pharmacy technicians. Some pharmacists tell our patients that they are “not a Suboxone pharmacy” and decline to stock it. This is a substantial barrier for our patients in small towns that may only have 1 local pharmacy. Some pharmacists will tell patients that they need to have daily observed doses or be on an opioid contract, even though it is indicated on the prescription to be dispensed every 30 days and prescribed under a pain paradigm. Several of our patients have decided not to proceed with induction or even stop therapy due to these obstacles. In short, systemic discrimination is keeping Canadians suffering from chronic pain from receiving a safer opioid management option.

However, there are moments in care when collaboration with our community colleagues have resulted in favourable patient outcomes. We call each pharmacy in advance as part of our clinic practice to facilitate the dispensing process of bup-nal, discuss the indication and induction method and give our contact information to answer any questions; in many cases, this has made the switch much easier for our patients. For example, one pharmacist called NS to let her know that our mutual patient was tapering down on bup-nal and was experiencing mild withdrawal symptoms. Together we were able to provide the support for our patient to continue with their therapeutic plan. Another positive example is when a patient had missed an appointment with our clinic and consequently ran out of bup-nal doses. The community pharmacist renewed her prescription until the patient’s next appointment. This reduced the risk of withdrawal and supported the patient through a difficult time. These examples highlight the absolute importance of pharmacists across the health care system, and our hope is that these same skills that pharmacists use to support patients in other areas of health care will translate more easily to bup-nal.

## Conclusion

In our professional opinion, bup-nal should be considered as an alternative to continuing full-mu agonist opioids, particularly in patients who have challenges tapering opioids and/or are at risk of opioid-related medical complications, including central nervous system or respiratory depression. There are significant challenges related to patient education, choosing an initial bup-nal dose, the actual conversion process and follow-up; consequently, these switches are typically conducted in chronic pain clinics. As pharmacists, we acknowledge the existence of stigma surrounding bup-nal, particularly in the context of the opioid crisis and bup-nal’s known clinical indication. Nevertheless, it is our responsibility to stay informed about the evolving evidence in opioid management and to put the patient’s best interest in the forefront. Through this commentary, we aim to raise awareness among pharmacists, empowering them to improve pain medication management for Canadians. In addition, we hope to foster a shift in attitudes, reducing the stigma and discrimination faced by individuals living with chronic pain.

Bup-nal is an effective and safe option for some individuals who are suffering from chronic pain, and pharmacists play a crucial role in supporting bup-nal accessibility while reducing stigma. Our commitment to supporting patients should remain unwavering, and by leveraging our expertise in pharmacology, pharmacokinetics and medication management, we can significantly contribute to enhancing the lives of those affected by chronic pain as well as providing support to both patients and their families. ■
